# Epithelial-to-Mesenchymal Transition and Cancer Invasiveness: What Can We Learn from Cholangiocarcinoma?

**DOI:** 10.3390/jcm4121958

**Published:** 2015-12-19

**Authors:** Simone Brivio, Massimiliano Cadamuro, Luca Fabris, Mario Strazzabosco

**Affiliations:** 1 School of Medicine and Surgery, University of Milan-Bicocca, Via Cadore 48, 20900 Monza, Italy; s.brivio3@campus.unimib.it (S.B.); massimiliano.cadamuro@gmail.com (M.C.); 2 Department of Molecular Medicine, University of Padua School of Medicine, Viale Colombo 3, 35131 Padua, Italy; luca.fabris@unipd.it; 3 Liver Center, Section of Digestive Diseases, Yale University, TAC Building, 333 Cedar Street, New Haven, CT 06520, USA

**Keywords:** cholangiocarcinoma, cholangiocyte, epithelial-to-mesenchymal transition, invasiveness, metastatization, tumor reactive stroma, cancer-associated fibroblast

## Abstract

In addition to its well-established role in embryo development, epithelial-to-mesenchymal transition (EMT) has been proposed as a general mechanism favoring tumor metastatization in several epithelial malignancies. Herein, we review the topic of EMT in cholangiocarcinoma (CCA), a primary liver cancer arising from the epithelial cells lining the bile ducts (cholangiocytes) and characterized by an abundant stromal reaction. CCA carries a dismal prognosis, owing to a pronounced invasiveness and scarce therapeutic opportunities. In CCA, several reports indicate that cancer cells acquire a number of EMT biomarkers and functions. These phenotypic changes are likely induced by both autocrine and paracrine signals released in the tumor microenvironment (cytokines, growth factors, morphogens) and intracellular stimuli (microRNAs, oncogenes, tumor suppressor genes) variably associated with specific disease mechanisms, including chronic inflammation and hypoxia. Nevertheless, evidence supporting a complete EMT of neoplastic cholangiocytes into stromal cells is lacking, and the gain of EMT-like changes by CCA cells rather reflects a shift towards an enhanced pro-invasive phenotype, likely induced by the tumor stroma. This concept may help to identify new biomarkers of early metastatic behavior along with potential therapeutic targets.

## 1. Introduction

Cholangiocarcinoma (CCA) is a primary liver cancer arising from the epithelial cells lining the intra and extrahepatic portions of the biliary tree (cholangiocytes). Although classically viewed as a relatively rare type of cancer, CCA is responsible for 10%–20% of the deaths related to primary liver malignancies, and its incidence has progressively increased starting from the early 1990s, at least for the intrahepatic variant. Unlike hepatocellular carcinoma, CCA does not usually develop within a background of chronic liver disease, which makes its diagnosis and treatment even more difficult [1]. In fact, CCA is most often diagnosed at an advanced stage, when intrahepatic or lymph node metastatic dissemination has already occurred, owing to the strong and early invasiveness of the tumor. Therefore, less than one-third of patients are eligible for radical surgery, which is, so far, the only treatment with curative intent, whereas most of them are merely located to palliative procedures. Unfortunately, the success of surgical resection is heavily threatened by the high rates of recurrence, resulting in a five-year survival of no more than 20%–30%. On the other hand, in the last few years liver transplantation has been proposed, but only for a highly selected subset of patients and by few specialized centers [2,3,4].

Overall, these gaps in knowledge support the need to better understand the molecular mechanisms underlying the invasive properties of CCA, with the ultimate goal to identify biomarkers of early metastatic behavior, useful for the decision-making process of patient allocation to the best treatment, and to develop targeted molecular therapies aimed at halting the metastatic spread of the tumor. In the last few years, growing attention has been drawn on epithelial-to-mesenchymal transition (EMT) as a mechanism promoting dissemination in several cancer cell types.

## 2. EMT Involvement in Cancer Cell Dissemination

Metastasis is a complex and continuously evolving process, which follows a specific sequence of events. In solid epithelial cancers, the cell metastatization follows four steps:

(1) Detachment from the highly-organized epithelial layer; this requires reducing cell-cell contacts and rearranging the cytoskeletal architecture, in favor of a motile phenotype;
(2) Impairment of the integrity of the basement membrane through active proteolysis, and then invasion of the surrounding stroma as strands or cords. Once in the tumor stroma, cells can efficiently cross-talk with multiple mesenchymal and inflammatory cell types, which in turn support their invasiveness;
(3) Dissemination at distance through the lymphatic and/or hematogenous circulation, taking advantage of the leaky neovasculature arising in the tumor microenvironment; and
(4) Engraftment at the distant sites by moving from the vessel lumen into the ectopic tissue parenchyma, wherein cells restart their deregulated proliferative program [5,6].

As a general concept, the metastatic cascade relies, at least partially, on the activation by the cancer cell of molecular programs typical of the mesenchymal lineage, as shown by profound changes in the expression of cytoskeletal and cell surface proteins, as well as by *de novo* expression of extracellular matrix (ECM)-degrading enzymes [7]. This observation led many researchers to speculate that carcinoma cells undergoing metastatization may somehow recapitulate the embryonic program of phenotypic conversion known as EMT [8]. During morphogenetic EMT, differentiated epithelial cells gradually acquire a full mesenchymal phenotype, characterized by the disassembly of cell junctions and the loss of cytokeratin filaments, with a concomitant gain of migratory functions, by which cells may leave their original localization within the epithelial sheets [9,10]. Through EMT, a number of key developmental events, such as embryo implantation, gastrulation and neural crest formation, can properly occur [11]. The EMT process is driven by a set of embryonic transcription factors, including Snail (Snail1), Slug (Snail2), Twist1/2 and ZEB1/2, which repress the expression of cytokeratins (K) and critical junction proteins, in particular E-cadherin, the molecular hallmark of the epithelial phenotype. On the same time, these transcription factors variably induce the expression of a range of mesenchymal markers, such as α-smooth muscle actin (α-SMA), vimentin, and S100A4. Moreover, increased production of ECM components, such as fibrillar collagen, and of matrix metalloproteinases (MMPs) is concurrently shown by cells undergoing EMT [12,13,14]. Activation of pro-EMT transcription factors is triggered by a broad spectrum of factors, encompassing cytokines and growth factors (such as transforming growth factor (TGF)-β1 and growth factors with affinity for receptor tyrosine kinases) [14,15], morphogenetic signals (namely Wnt, Notch and Hedgehog (Hh) signaling) [16], and post-transcriptional gene regulator microRNAs (miRNA) (e.g., miR-200 family members) [17]. These triggering factors can be released as effect of several disease mechanisms, in particular chronic inflammation, hypoxia and autophagy, most of which may be involved in malignant transformation [18,19,20].

## 3. Evidence for EMT in Human Carcinomas

The ability of tumor cells to express at different levels some mesenchymal properties is largely recognized. These include the loss of cell-to-cell adhesion (usually modulated by the E-cadherin to N-cadherin switch), alterations in cell polarity (from apical-basal to front-rear) and cell shape (from cobblestone-like to spindle-like), expression of mesenchymal biomarkers, such as vimentin and S100A4, and proteolytic activities [21,22]. Notably, cells expressing EMT biomarkers are more frequently localized at the invasive front rather than in the bulk of the tumor [9,23]. Nonetheless, EMT signatures (that we would rather call “transitional” properties) have been widely reported in circulating tumor cells [24,25], thus highlighting the concept that these ”transitional” properties identify a subset of tumor cells more prone to be engaged in invasive processes. Furthermore, many clinical studies correlated the expression of EMT features with an increased metastatic potential and a poor clinical outcome in several carcinomas, including breast [26,27], pancreatic [28], gastric [29], colorectal [30], and lung cancer [31]. This clinical evidence is consistent with experimental data, showing the ability of TGF-β1, Snail and Twist, to induce the expression of mesenchymal features in human cultured cancer cell lines, *in vitro* [32,33,34,35], and to enhance their metastatic potential in xenograft models [36,37,38]. Notwithstanding, the actual relevance of EMT in human tumor progression still remains uncertain. In this regard, CCA is an epithelial cancer type with several peculiarities particularly suitable to address this issue.

## 4. Expression of EMT Features in CCA and Underlying Mechanisms Involved

Phenotypic features of EMT, including up-regulation of vimentin, S100A4, Snail and Twist, in conjunction with down-regulation of E-cadherin and of membranous β-catenin, have been observed in neoplastic bile ducts [39]. Most of them frequently correlated with tumor progression and more severe prognosis [40,41,42,43]. For example, low expression of E-cadherin in CCA tissues, significantly associated with the presence of metastasis, and tended to correlate with a shorter survival time [44]. In this context, our group recently showed that S100A4, when expressed in the nucleus of neoplastic bile ducts, is a strong predictor of increased invasiveness and metastatization in CCA patients. Moreover, we demonstrated that relevance of nuclear S100A4 went well beyond that of a mere surrogate marker of invasiveness, as it was functionally able to promote the acquisition of a metastatic phenotype. Indeed, human CCA cells harboring nuclear expression of S100A4 displayed increased metastatic abilities when xenotransplanted into SCID mice, compared with CCA cells not expressing S100A4 in the nucleus. Mechanistic relevance of S100A4 was further supported by *in vitro* studies showing that down-modulation of nuclear S100A4 in CCA cells by lentiviral silencing or by pharmacological treatment with paclitaxel significantly reduced their motility and invasive functions. These effects were associated with a reduction in the activities of Rho-A and Cdc42, small Rho GTPases known to affect the directionality of cell migration, and of MMP-9, a pivotal proteolytic enzyme in cancer invasiveness [7,45]. Factors modulating the ability of CCA cells to express different “transitional” features are gradually emerging and, potentially, may provide a target of therapeutic intervention to halt CCA invasiveness. Under their effect, the up-regulation of EMT markers goes hand in hand with increased cell invasiveness, *in vitro*.

### 4.1. Cytokines, Growth Factors and Morphogens Promoting EMT

Multiple soluble factors are able to induce a “transitional” phenotype in cultured CCA cells. They are summarized in Table 1. These factors are variably released into the tumor microenvironment by different cell sources, supporting involvement of both autocrine and paracrine mechanisms. In fact, besides tumoral cells, cells recruited within the tumor reactive stroma (TRS), closely aligning with the neoplastic ducts, may be also strong producers of EMT inducers. The TRS is a highly-specialized mesenchymal compartment hosting several cell types, such as cancer-associated fibroblasts (CAFs) and tumor-associated macrophages (TAMs), which provide cancer cells with a broad range of cues directly stimulating their malignant behavior [46]. In fact, CAFs secrete high levels of TGF-β, IL-6, SDF-1, EGF, and FGF, whereas TAMs variously produce TGF-β, TNF-α, IL-6, and EGF. Moreover, either cell types may release several MMPs, able to trigger EMT changes by cleaving essential cell adhesion molecules on the surface of cancer cells [46,47]. Human CCA cells cultured with conditioned media harvested from activated macrophages actually showed a strong down-regulation of E-cadherin and K-19, in conjunction with an up-regulation of S100A4 and MMP-9 [48], and with increased migratory properties, *in vitro* [49]. Similarly, SDF-1 produced by CAFs, was reported to promote the invasiveness of cultured CCA cells, marked by *de novo* expression of vimentin, and decreased expression of E-cadherin and membranous β-catenin [50]. Similar evidence was reported in other cancers featuring an abundant desmoplasia, such as breast [51] and colorectal [52] cancer. Of note, the hypoxic microenvironment typically featuring CCA has been proposed as an important stressor exacerbating the release of EMT inducers [53]. For example, hypoxia may induce the secretion of the multifunctional peptide adrenomedullin (ADM) by CCA cells. ADM overexpression was observed in neoplastic bile ducts, and, *in vitro,* it associated with the induction of tumor cell migration and invasion via EMT [54].

**Table 1:** Soluble factors inducing a “transitional” phenotype in cultured CCA cells.

EMT Inducer	References
Inflammatory cyto/chemokines	
TGF-β1	[55,56,57,58]
TNF-α	[59]
IL-6	[60]
HMGB1	[61]
SDF-1	[50]
Growth factors	
EGF	[62,63]
FGF-19	[64]
Morphogens	
Notch1/Sox9	[65,66,67]
Sonic Hh	[68]

Transforming growth factor β1, TGF-β1; tumor necrosis factor α, TNF-α; interleukin 6, IL-6; high-mobility group box 1, HMGB1; stromal cell-derived factor 1, SDF-1; epidermal growth factor, EGF; fibroblast growth factor 19, FGF-19; Hedgehog, Hh.

Several epigenetic mechanisms, including DNA methylation and histone post-translational modifications, have been hypothesized to regulate the expression of EMT-related signatures, in line with the concept of pronounced plasticity of tumoral cells that may dynamically adapt to various microenvironmental stimuli [69]. For example, Snail-induced E-cadherin repression is mediated, at least in part, by the direct recruitment of both histone deacetylases and DNA methyltransferases at the E-cadherin promoter [70,71]. The ability to induce epigenetic changes in co-cultured cancer cells has been reported in CAFs from gastric, ovarian and breast cancer [72,73,74]. In particular, CAFs from gastric cancer induced in tumoral cells a down-modulation of E-cadherin coupled with increased migratory functions through a DNA methylation-dependent inactivation of miR-200b (see below), which led to ZEB1/2 activation [72]. It is tempting to speculate that similar epigenetic events may also occur in CCA, possibly driven by interactions with the TRS components [12,46], an issue worthy of particular attention by future studies.

### 4.2. miRNAs Promoting EMT

miRNAs are endogenous non-coding RNAs of 20–25 nucleotides, which regulate mostly negatively gene expression by directly interacting with target mRNAs, thus inhibiting their translation and/or promoting their cleavage. Besides their involvement in physiological processes, increasing evidence suggests that several miRNAs play a pathogenic role in human cancers, as highlighted by the differential expression profiles between tumor and normal tissues. Indeed, most of miRNA genes are located within cancer-associated genomic regions [75]. Several miRNAs have been found to be deregulated in human CCA cell lines and/or tissues, wherein they affect a wide range of processes related to tumor biology, such as proliferation (e.g., miR-21, miR-26a, miR-31, miR-421, miR-494), apoptosis (e.g., miR-21, miR-25, miR-29b, miR-31, miR-204, miR-320), migration (e.g., miR-21, miR-138, miR-200b, miR-220c, miR-376c, miR-421), differentiation (e.g., miR-200b, miR-373), angiogenesis (e.g., miR-101) and chemoresistance (e.g., miR-21, miR-29b, miR-200b, miR-205, miR-221) [76,77]. In this regard, recent studies have shown that inactivation of specific miRNAs may also induce an EMT phenotype. Those found inactivated in CCA cells and their EMT-related target genes are illustrated in Table 2. Among them, neural cell adhesion molecule (NCAM) is a surface glycoprotein expressed by immature cholangiocytes and functionally linked to EMT, and Smad4 is a common downstream effector of the TGF-β pathway. Conversely, miR-21 promotes EMT changes when aberrantly activated [78]. Noteworthy, the expression of miR-214, miR-204 and miR-34a was significantly reduced in CCA specimens, compared with normal tissues, and their down-regulation closely correlated with CCA metastasis.

**Table 2:** miRNAs whose inactivation leads to EMT induction.

miRNA	Target Gene	References
miR-214	Twist	[79]
miR-204	Slug	[80]
miR-200c	ZEB1/2; NCAM	[81]
miR-34a	Smad4	[82]

Neural cell adhesion molecule, NCAM.

### 4.3. Oncogenes and Tumor Suppressor Genes Regulating EMT

An altered expression of both oncogenes and tumor suppressor genes may also entail EMT changes promoting CCA metastatization. Among oncogenes, the zinc finger transcription factor spalt-like transcription factor 4 (SALL4) [83], and the transcriptional co-activator yes-associated protein (YAP) [84], are found to play a relevant role in CCA. SALL4 ability to induce mesenchymal properties relates to its well-documented interactions with TGF-β and Wnt signaling pathways [85], whereas YAP triggers EMT by increasing the expression of the oncoprotein gankyrin in an AKT-dependent manner [84]. With respect to tumor suppressor genes, two recent studies have shed light on the role of the ubiquitin ligase F-box and WD repeat domain-containing 7 (FBXW7), and of the protein kinase mitogen-activated protein 3 kinase 4, as negative regulators of mTOR/ZEB1 and p38/NF-κB/Snail pathways, respectively [86,87]. Importantly, the role of YAP and FBXW7 in CCA invasiveness was confirmed also *in vivo* by xenograft models, where YAP overexpression or FBXW7 knockdown led to an increased cancer dissemination. In gallbladder cancer (GBC), the most common malignancy of the biliary tract, pathologically distinct from CCA, but characterized by a similar invasive phenotype, occurrence of EMT-like changes has been recently linked to the newly identified tumor suppressor gene *N*-myc downstream-regulated gene (NDRG)-2. GBC cells with loss of NDRG2 expression showed EMT-like features associated with enhanced migration and invasiveness *in vitro*, and tumor growth and metastasis *in vivo*. The study elegantly unraveled the molecular mechanism activated by the loss of NDRG2 expression, leading to the up-regulation of MMP-19, which, in turn, directly promoted the expression of Slug at the transcriptional level, ultimately responsible for EMT-like changes. Furthermore, MMP-19-induced Slug increased the expression of a receptor tyrosine kinase, Axl, which maintained Slug expression through a positive feedback loop, and stabilized EMT of GBC cells. Altogether, these findings unveil a novel role for MMPs, acting as EMT inducing transcriptional regulators [88].

### 4.4. Disease Mechanisms Inducting the “Transitional” Phenotype

Multiple disease mechanisms underlying CCA carcinogenesis and progression have been associated with EMT. Ability of chronic inflammation to induce EMT changes relies on the effects of cytokines, chemokines, and growth factors widely released in the portal tract by inflammatory cells (neutrophils, lymphocytes, macrophages, myofibroblasts) in many cholangiopathies (*i.e.*, primary biliary cirrhosis, primary sclerosing cholangitis, congenital hepatic fibrosis), as previously outlined. Furthermore, some etiologic agents may induce EMT because of their intrinsic liver damaging activity. For example, the chronic hepatitis C virus (HCV) infection is an established risk factor for CCA [1]. In human CCA specimens, the positive expression of the HCV core protein (HCVc) associated not only with lymph node metastasis, but also with EMT features (increased expression of vimentin, fibronectin and *N*-cadherin, and decreased expression of E-cadherin). Consistently, HCVc promoted “transitional” changes in cultured CCA cells, including enhanced motility and invasion, by increasing the expression and/or activity of lysyl oxidase-like 2, which prevented Snail degradation [89,90].

Autophagy is emerging as a key regulator of cell invasion in a number of human cancers, triggered by various stimuli, such as nutrient deprivation and hypoxia [91,92]. A recent study showed that starvation-induced autophagy enhanced the invasive properties of CCA cells, and, in parallel, autophagy inhibition by chloroquine significantly abrogated the TGF-β1-induced cell invasion, thus arguing for a possible autophagy-dependent EMT regulation in CCA. Indeed, in human CCA specimens, the expression of the autophagy-related protein activating molecule in Beclin1-regulated autophagy positively correlated with the expression of Snail as well as with lymph node metastasis [93].

## 5. EMT and CAFs Generation: Insights from CCA

In addition to supporting the pro-invasive functions of tumoral epithelial cells, EMT has been claimed as a potential ancillary mechanism generating CAFs, which would represent a full mesenchymal switch [8,94]. To address this specific issue, we developed an *in vivo* xenograft model of CCA enabling us to trace the fate of cancer cells by a dual tracking system. An enhanced green fluorescent protein (EGFP)-expressing human male CCA cell line (EGI-1) displaying an EMT phenotype was xenografted by intraportal injection into a severe combined immune deficiency (SCID) male mouse, and dual immunofluorescence for EGFP (CCA cell marker) and α-SMA (CAF marker) was performed on liver tumors. In this model, cancer cells undergoing a complete EMT would be expected to co-express both markers. However, although engrafted tumors faithfully reproduced the native CCA characteristics, as shown by the abundant TRS surrounding the EGFP-positive, EGI-1-derived bulk, coincident labeling between EGFP and α-SMA was never observed. Moreover, a FISH analysis revealed that the human Y chromosome specific probe was not expressed by α-SMA-positive cells (which on the contrary, expressed the murine Y chromosome), but only by EGFP-positive CCA cells. In accordance with these data, human CCA sections did not display co-expression of the cholangiocyte lineage marker K7 and α-SMA. Taken together, these data strongly argue that in CCA, CAFs are not generated through an EMT of cancer cells, *in vivo* [39].

## 6. CCA as Model to Redefine the Concept of EMT in Cancer Invasiveness

Many studies suggested that EMT plays a crucial role in promoting epithelial cancer invasiveness, based on the concept that several EMT features are coherent with an invasive phenotype, and indeed, they can be reproduced by *in vitro* experiments. However, in CCA, a complete transition of epithelial cancer cells toward a mesenchymal phenotype is not found *in vivo* [39]. All the well-recognized mesenchymal features commonly displayed by neoplastic cholangiocytes, although strongly involved in CCA dissemination, are more consistent with the concept of “transitional” changes leading to an invasive phenotype, but developing in the context of a preserved native epithelial identity [46]. The general assumption that the gain or loss of expression of one or more molecules typically serving as lineage biomarkers necessarily reflects a large-scale gene expression reprogramming is appealing, but questionable. In CCA, the presence of a “transitional” phenotype may instead represent the synergic effect between an ongoing tumoral dedifferentiation process, regulated by stochastic mutational events, and the influence of paracrine signals originating from the TRS [9,95,96]. When the resulting changes in gene expression are such that cancer cells gain a more malignant phenotype endowed with pro-invasive features, those cells would then likely become overrepresented through a process of Darwinian selection [5]. However, these phenotypic dynamics are markedly different from the finely-regulated lineage conversion program typically occurring in embryonic cells undergoing EMT during organogenesis.

It is worth underlining that a highly malignant behavior associated with EMT by several studies is that of the cancer stem cell (CSC) endowed with unlimited self-renewal capabilities, heightened resistance to apoptosis and strong chemoresistance [97]. Shuang *et al.* [98] reported that TGF-β1-induced EMT provides CCA cells with a range of stem cell-like features, including expression of pluripotency transcription factors (Sox2 and Oct3/4) and enhanced resistance to chemotherapeutic drugs. Consistently, CCA cells constitutionally expressing the CSC biomarker aldehyde dehydrogenase (ALDH), showed reduced E-cadherin expression, and increased expression of vimentin, fibronectin and *N*-cadherin, compared with ALDH-negative cells [98]. This intimate link between EMT and stemness may be a pivotal factor promoting metastatic colonization. Indeed, the concept that only metastasizing cells endowed with self-renewal capabilities are responsible for ectopic tumor dissemination [5] is emerging. Interestingly, co-expression of stem cell and EMT properties has also been reported in cell populations isolated from early human fetal liver [99], suggesting that the “transitional” changes undergone by neoplastic cholangiocytes may overall represent an aberrant reactivation of an embryonic behavior.

## 7. Conclusions

**Figure 1:**
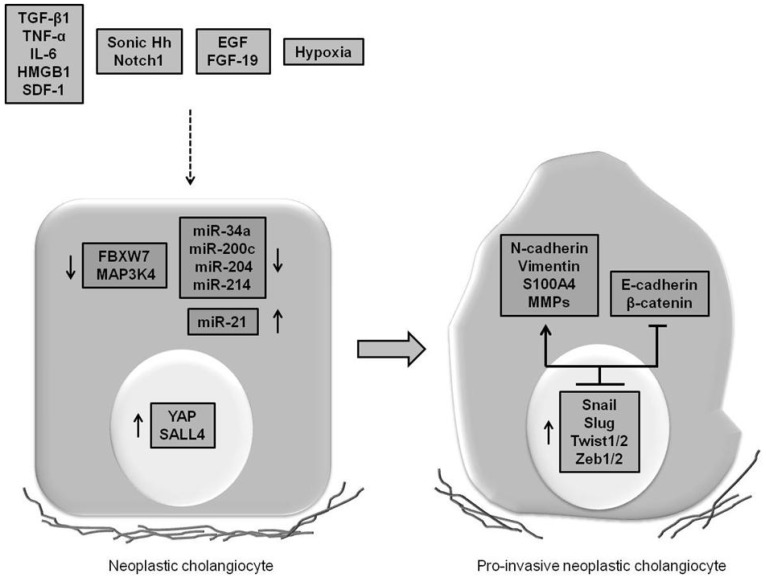
Autocrine and paracrine signals and intracellular stimuli orchestrate the switch towards a “transitional” phenotype during neoplastic transformation. The emergence of mesenchymal traits in CCA cells (up-regulation of *N*-cadherin, vimentin, S100A4 and metalloproteinases, along with down-regulation of E-cadherin and membranous β-catenin) is driven by a set of embryonic transcription factors (Snail, Slug, Twist1/2, and Zeb1/2), whose expression is induced by both soluble factors released in the tumor microenvironment (cyto/chemokines, growth factors, and morphogens) and mutational events affecting the activity of miRNAs, oncogenes, and tumor suppressor genes. These “transitional” changes allow cancer cells to reduce intercellular adhesion and dismantle the basement membrane, resulting in a motile phenotype. Transforming growth factor β1, TGF-β1; tumor necrosis factor α, TNF-α; interleukin 6, IL-6; high-mobility group box 1, HMGB1; stromal cell-derived factor 1, SDF-1; epidermal growth factor, EGF; fibroblast growth factor 19, FGF-19; Hedgehog, Hh; F-box and WD repeat domain-containing 7, FBXW7; mitogen-activated protein 3 kinase 4, MAP3K4; yes-associated protein, YAP; spalt-like transcription factor 4, SALL4; metalloproteinase, MMP.

The aggressiveness of CCA is associated with the expression by neoplastic ducts of “transitional”/pro-invasive features, some of which have also a strong prognostic value. These are the result of paracrine signals originating from the stromal compartment, which closely accompanies the growth of tumoral ducts, acting in concert with signaling perturbations arising in the cancer cells themselves (Figure 1). However, these phenotypic changes do not reflect a full conversion of CCA cells towards a mesenchymal phenotype, which potentially would lead to the generation of the reactive stroma, a feature particularly abundant in CCA. Nonetheless, the gain of EMT-like features by neoplastic cholangiocytes may have profound implications for the management of CCA patients, by identifying biomarkers serving as prognostic factors and/or predictors of treatment response, as it is the case with nuclear S100A4, and by providing novel molecular therapeutic targets. Notably, some EMT-interfering agents, such as inhibitors of TGF-β signaling, TGF-β type I receptor, and EGF receptor, have been recently tested in clinical trials in patients with carcinomas with promising effects [100].
